# Measures and Limits of Models of Fixation Selection

**DOI:** 10.1371/journal.pone.0024038

**Published:** 2011-09-12

**Authors:** Niklas Wilming, Torsten Betz, Tim C. Kietzmann, Peter König

**Affiliations:** Institute of Cognitive Science, University of Osnabrück, Osnabrück, Germany; The University of Plymouth, United Kingdom

## Abstract

Models of fixation selection are a central tool in the quest to understand how the human mind selects relevant information. Using this tool in the evaluation of competing claims often requires comparing different models' relative performance in predicting eye movements. However, studies use a wide variety of performance measures with markedly different properties, which makes a comparison difficult. We make three main contributions to this line of research: First we argue for a set of desirable properties, review commonly used measures, and conclude that no single measure unites all desirable properties. However the area under the ROC curve (a classification measure) and the KL-divergence (a distance measure of probability distributions) combine many desirable properties and allow a meaningful comparison of critical model performance. We give an analytical proof of the linearity of the ROC measure with respect to averaging over subjects and demonstrate an appropriate correction of entropy-based measures like KL-divergence for small sample sizes in the context of eye-tracking data. Second, we provide a lower bound and an upper bound of these measures, based on image-independent properties of fixation data and between subject consistency respectively. Based on these bounds it is possible to give a reference frame to judge the predictive power of a model of fixation selection . We provide open-source python code to compute the reference frame. Third, we show that the upper, between subject consistency bound holds only for models that predict averages of subject populations. Departing from this we show that incorporating subject-specific viewing behavior can generate predictions which surpass that upper bound. Taken together, these findings lay out the required information that allow a well-founded judgment of the quality of any model of fixation selection and should therefore be reported when a new model is introduced.

## Introduction

A magnificent skill of our brain is its ability to automatically direct our senses towards relevant parts of our environment. In humans, the visual capacity has by a large margin the highest bandwidth, making directing our eyes towards salient events the most important method of selecting information. We sample the visual input by making targeted movements (saccades) to specific locations in the visual field, resting our gaze on these locations for a few hundred milliseconds (fixations). Controlling the sequence of saccades and fixation locations thereby determines what parts of our visual environment reach our visual cortex, and contingently conscious awareness. Understanding this process of information selection via eye movements is a key part of understanding our mental life.

A common approach to investigate this process has been to use computational models that predict eye movements to gain insights on how the brain solves the problem of determining where in a scene to fixate [1]–[7]. The similarity of empirical eye-tracking data and model predictions is then used as an indication of how well the model captures essential properties of the fixation selection process. For this chain of reasoning, i.e. for drawing inferences about the workings of the brain, it is highly relevant how the quality of a model of fixation selection is measured. Furthermore, if different models are to be compared and judged, there needs to be an agreed upon metric to make this comparison possible. Of equal importance for model comparisons is the data set that is being used as ground truth. Different data sets might be more or less difficult to predict, which confounds a potential model comparison across different studies. In this article, we investigate metrics for evaluating models of fixation selection, and methods to quantify how well models of fixation selection can score on a specific data set. This leads to a framework for evaluating and comparing models.

Before we can discuss how measures and data set influence the evaluation, we have to be clear about what models of fixation selection actually predict. Even though the ultimate goal of the model may be to predict fixation locations, the actual mechanism of fixation selection is usually not addressed in detail. Instead the focus is on computing a topographic representation of how strongly different parts of the image will attract fixations. Classically, each region in an image is assigned a so-called salience value based on low-level image properties (e.g. luminance, contrast, color) [1]–[7]. The topographic representation of the salience values for all image regions is known as the salience map. Some models furthermore incorporate image-independent components, like the fact that observers tend to make more fixations in the center of a screen than in the periphery regardless of the presented image, known as a spatial (or central) bias [7]–[10]. Other forms of higher level information that have been used in models of fixation selection are task-dependent viewing strategies, information about face-locations and search-target similiarity [11]–[14]. However, even in those models the important output is a map of fixation probabilities. Thus, in accordance with the focus on this approach in the modeling literature, we restrict our analysis to the evaluation of models that generate a salience map. Since the empirical data that these salience maps have to be evaluated against are not maps themselves, but come in the form of discrete observations of fixation locations, it is not obvious a priori how to judge the quality of such a model.

In the first part of this article, we therefore review different commonly used evaluation measures. We define properties that are desirable for evaluation measures and provide evidence that many commonly used measures lack at least some of these properties. Because no single measure has all of the desirable properties, we argue that reporting both the Area Under the receiver-operating-characteristic Curve (AUC) for discriminating fixated from non-fixated locations, and the Kullback-Leibler divergence (KL divergence) between predicted fixation probability densities and measured fixation probability densities, gives the most complete picture of a model's capabilities and facilitates comparison of different models.

In the second part of this work, we turn to properties of fixation distributions and examine what impact they have on model evaluation and comparison. Our aim is to formalize the notion of how difficult a data set is to predict, which will facilitate comparisons between models that are evaluated on different datasets . We use the image- and subject-independent distribution of fixation locations (spatial bias) to establish a lower bound for the performance of attention models that predict fixation locations. The predictive power of every useful model should surpass this bound, because it quantifies how large evaluation scores can become without knowledge of the image or subject to be predicted . Complementary to this, we use the consistency of selected fixation locations across different subjects (inter-subject consistency) as an upper bound for model performance, following [3], [12], [13], [15]–[17]. The reliability of these bounds depends on how well they can be estimated from the data being modeled. We therefore provide a detailed investigation of the spatial bias as well as inter-subject consistency, and their dependence on the size of the available data set. This establishes a reference frame that allows judging whether improvements in model performance are informative of the underlying mechanism and facilitates model comparison.

Finally, we examine the conditions under which the proposed upper bound holds by turning to a top-down factor that has so far been neglected in the literature. We show that incorporating subject idiosyncrasies improves the prediction quality over the upper bound set by inter-subject consistency. This should be interpreted as a note of caution when using our proposed bounds, but does not call into question their validity in the more general and typical case of modeling the viewing behavior of a heterogeneous group of subjects.

## Results

### Measures of model performance

In this section, we review commonly used measures for the evaluation of models of fixation selection. Our aim is to investigate, on a theoretical basis, what the advantages and disadvantages of different measures are and to identify the most appropriate measure for model evaluation. To reach this aim, we choose a four step approach. First, we establish a list of desirable properties for evaluation measures. Second, we identify commonly used measures in the literature and describe how they compare model predictions to eye-movement data. Third, we assess how the measures fare with regard to the desirable properties. Justified by this, we recommend the use of the AUC. Finally, we elucidate the effect of pooling over subjects and conclude that in some circumstances, KL-divergence is a more appropriate measure.

#### Desirable Properties for evaluation measures

Evaluation scores of a model of fixation selection will at some point be used to compare it to other models. Such comparisons are not only difficult because different data sets are being used, but also because the interpretation of evaluation measures can be difficult. Informed by our own modeling work and by teaching experience, where several points repeatedly obstructed the comparison of different models, we define two properties that help to interpret evaluation scores:

- Few parameters: The value of an evaluation measure ideally does not depend on arbitrary parameters, as this can make the comparison of models difficult. If parameters are needed, meaningful default values or a way of determining the parameters are desirable.
- Intuitive scale: A good measure should have a scale that allows intuitive judgment of the quality of the prediction. Specifically, a deviation from optimal performance should be recognizable without reference to an external gold standard.

Models of fixation selection are usually evaluated against eye-tracking data, which is typically very sparse in relation to the size of the image that is being viewed. It is therefore desirable for an evaluation measure to give robust estimates based on low amounts of data:

- Low data demand: During a typical experiment, subjects can usually make only a relatively small number of saccades on a stimulus. Thus, an ideal measure should allow for a reliable estimate of the quality of a prediction from very few data points.
- Robustness: A measure should not be dominated by single extreme or unlikely values. Consider, for example, that the prediction of a fixation probability distribution consists of potentially several million data points. The result of the prediction of a single data point should not have a large impact on the overall evaluation. A measure should also be able to deal with the kinds of distributions typically occurring in eye-tracking data. A fixation density map (see sec∶mam: sec∶fdm) is usually not normally distributed but, due to its sparseness, dominated by the presence of many very unlikely events.

The properties presented here aim at ensuring that an evaluation measure is suitable to deal with eye-tracking data and to ensure that an evaluation score can be meaningfully interpreted. The list is not necessarily exhaustive, but we argue that any exhaustive list would have to contain these properties.

#### Existing measures

To identify commonly used measures, we sought articles that present or compare salience models which operate on static images of natural scenes. We used the Google Scholar bibliographic database (scholar.google.com) to search for articles that were published after the year 2000 and contain the words “eye”, “movement”, “model”, “salience”, “comparison”, “fixation”, “predicting” and “natural” somewhere in the text. This list of key-words was selected because omitting any one of them disproportionately increases the number of results unrelated to models of human eye movements. The search was performed on June 28, 2011. We manually checked the first 200 articles for evaluations of salience models on static natural scenes. In the resulting 25 articles [1]–[8], [10]–[27] eight different measures are used to compare eye-tracking data to predictions of fixation locations.

We sort the seven different measures into three groups, based on the comparison they perform. The three measures in the first group , chance-adjusted salience, normalized scan-path salience and the ratio of medians, compare the central tendency of predicted salience values at fixated locations with salience values at non-fixated locations. The second group, comprising 80th percentile, AUC and the naïve Bayes classifier, treats the salience map as the basis for a binary classification of locations as either fixated or non-fixated and evaluates the classification performance. The third group includes the KL-divergence and the Pearson product moment correlation coefficient. For these measures, the model output is interpreted as a fixation probability density, and the difference between this and a density estimated from actual fixation data is computed.

- *Chance-adjusted salience (* 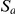 *)* [5] is the difference between the mean salience value of fixated locations on an image and the mean salience value of the viewed image. Thereby, if values are larger than 0, salience values at fixated locations are above average.
- *Normalized scan-path salience (NSS)* [6] is the mean of the salience values at fixation locations on a salience map with zero mean and unit standard deviation.
- The *ratio of medians* [27] compares the salience values at fixated locations to the salience at random control points. The salience value of a location is determined by finding the maximum of the salience map in a circular area of radius 5.6 degree around that location. The median salience at fixated locations and the median salience of a set of random control points on the same image are computed for each image. The ratio of both medians is used as evaluation measure.
- The 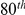 *percentile measure* [14] reports the fraction of fixations that fall into the image area that is covered by the top 20% of salience values. It therefore reports the true positive rate of a classifier that uses the 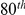 percentile of the salience distribution as a threshold. The selected area covers, by definition, 20% of the image, which is therefore the expected value for a random prediction.
- The *area under the receiver-operating-characteristics curve (AUC)* [10] describes the quality of a classification process. Here, the classification is based on the salience values at fixated and non-fixated image locations. All locations with a salience value above a threshold are classified as fixated. The AUC is the area under the curve that plots the true positive rate against the false alarm rate for all possible thresholds (the receiver operating characteristic). As the threshold is continuously lowered from infinity the number of hits and false alarms are both increasing. When the salience map is useful, the hits will increase faster than the false alarms. With still lowering threshold the latter will catch up and the fraction of hits and false alarms both reach 1 (100%). The AUC gives an estimate of this trade-off. An area of 1 indicates perfect classification, 100% hits with no false alarms. An area of 0.5 is chance performance. See [28] for an introduction to ROC analysis.
- The *percent correct of a naïve Bayes classifier* [8] that distinguishes between salience values at fixated and non-fixated locations can be used as a model evaluation measure. The classifier is trained by estimating the probability distributions 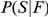 and 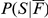 , where S refers to the salience value of a point and F signals if the point was fixated or not, on a subset of the data. Unseen data points are classified as fixated based on their salience if 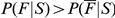. The percent correct score is computed in a cross-validation scheme such that all data points are classified as part of the test set once.
- The *Kullback-Leibler divergence (* 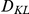 *)* [2], [29] is a measure of the difference between two probability distributions. In the discrete case it is given by:
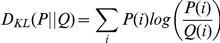
In the case of salience map evaluations, P denotes the true fixation probability distribution and Q refers to the model's salience map that is a 2D probability density function. For every image location the true fixation probability is divided by the model fixation probability and the logarithm of this ratio is weighted with the true fixation probability of the location. Therefore, locations that have a high fixation probability are emphasized in the 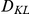 values. The 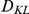 is a non-symmetric measure (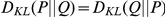 does not hold for all 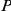 and 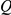). This is irrelevant for model evaluation, but becomes relevant when it is not clear what the true probability is, e.g. for evaluating inter-subject variability. In this case, a symmetric extension of 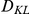 can be obtained by 
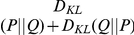
.
- The *Pearson product-moment correlation coefficient (correlation)* [13], [25] is a measure of the linear dependence between two variables. The correlation coefficient between two samples is given by:
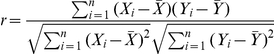
where 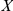 and 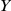 are the two variables, and 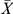 and 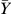 are the sample means. Evaluating models of fixation prediction with this measure requires a little conceptional gymnastics. If the values in a prediction map are interpreted as observations of variable 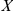, and the values in the empirical fixation probability distribution at the same pixel locations are interpreted as observations of variable 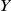 with the same index, the correlation coefficient between prediction and ground truth can easily be computed. The Pearson product moment correlation coefficient is bounded between 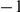 for predictions that are the inverse of the ground truth (ground truth multiplied with a negative number, plus or minus any number), and 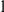 for perfect predictions. A value of 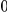 indicates that there is no linear relation between the prediction and the empirical fixation density.

#### Evaluation of measures with respect to the described properties

Having proposed a list of desirable properties and introduced a number of different measures, we can now examine how these measures cope with the requirements and what aspect of the prediction they evaluate. For an overview, please see Table 1.

**Table 1:** Summary of described evaluation measures.

	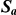	NSS	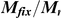	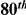	AUC	naïve Bayes	KL	correlation
Intuitive Scale	−	0	−	+	+	+	−	0
Few Parameters	+	+	−	−	+	−	−	−
Robustness	−	−	+	+	+	−	−	−
Low data demand	+	+	+	+	+	−	−	−

The table shows a summary of the evaluation measures and their performance with regard to the desirable properties described above. ‘+’ indicates that the measure exhibits the property, while ‘0’ and ‘−‘ indicate that the measure is neutral w.r.t. to the property or does not exhibit it.

- *Few parameters:* There are three measures that do not have parameters: 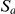, NSS and AUC. The ratio of medians is dependent on the radius that is used for selecting a salience value for a fixation. Although there may be reasons for choosing one value over another, this parameter is essentially arbitrary. The percentile chosen for the 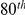 percentile measure is completely arbitrary; it might as well be the 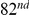 percentile. For the naïve Bayes classifier , the correlation and the KL-divergence, it is necessary to estimate probability distributions, which in the simplest case depends on the binning used. The naïve Bayes classifier furthermore requires the specification of the number of cross-validation runs.
- *Intuitive scale:* 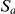 does not have an intuitive scale since the mean and range of a salience map are arbitrary and both influence the scale. The ratio of medians method is also not intuitive as it is not obvious how the resulting scores are to be interpreted. What does it mean that salience at fixated locations is 1.3 times higher than at random locations? What would it mean if it were 1.4 times higher instead? The interpretation of KL-divergence scores is also difficult for similar reasons. NSS has a rather intuitive scale because it uses the standard deviation of the salience map as its unit. All three classifying measures (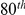 percentile, AUC, naïve-Bayes) are bounded, which should make their score easy to interpret by comparing the model score to the theoretical maximum. However, when using eye-tracking data, the categorization of points into the classes ‘fixated’ and ‘non-fixated’ is non-trivial. Strictly speaking, there are no non-fixated points: If we just record data long enough, there is no principle reason why a specific point on the screen cannot be fixated. Thus, any method for selecting non-fixated and fixated points will produce overlapping sets, which cannot be perfectly separated. In turn, no classifier can reach its theoretical maximum score in this task. In sec∶mam: sec∶theoretical_auc we show how to approximate the actual theoretical maximum score of the AUC, given a set of fixations. Despite these considerations, the meaning of classification performance (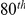 percentile, naïve Bayes) is straightforward. The meaning of the AUC is not as intuitive but also allows to quickly assess the quality of a model. The interpretation of correlation scores is rather intuitive: scores are bounded from both sides and can be interpreted as the linear dependence between prediction and ground truth. However, interpretation of a specific correlation value becomes less trivial if the actual dependence structure is not linear. In that case, which is typical for fixation data, the measure can be misleading when interpreted as if the condition of linearity was fulfilled.
- *Low data demand:* The three methods that require probability density functions, KL-divergence , correlation and naïve Bayes classifier, require a lot of data to form accurate estimates of the necessary probability distributions. In contrast, all other methods use only the fixated locations as positive instances and can in principle be computed on very few data points.
- *Robustness:* 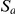 uses the mean to summarize information about salience values at fixation locations. Since the mean is not robust against outliers, neither is 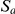. NSS also uses the mean, but first normalizes the salience map to zero mean and unit standard deviation. Thus, extreme outliers will have a weaker effect than for 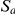, but still influence the result. The ratio of medians uses the median as a descriptive statistic of salience at fixated and control points. This ensures that extreme outliers have no negative effect. The naïve Bayes classifier is not by definition robust against outliers, as its robustness depends very much on how the necessary probability distributions are estimated. If simple bin counting is used it is not robust against outliers. Similar arguments hold for the KL-divergence and the correlation , where the true fixation probability distribution has to be estimated from the data.

In summary, our evaluation shows that there are large differences in the suitability of the different measures when it comes to evaluating models of fixation selection . 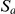, NSS and the ratio of medians are not intuitive to interpret and/or not robust. From the three classification measures, the AUC appears to be most favorable. It improves on the 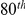 percentile measure by removing the arbitrary parameter and by including false alarms into the analysis. The naïve Bayes approach needs more data than is often available and the estimation of probability density maps is non-trivial. Correlation and KL-divergence need much data and require the estimation of density functions. Additionally, KL-divergence is not easy to interpret, but has a sound theoretical basis when the comparison of probability densities is concerned . The AUC stands out on the properties we have outlined. Based on our defined requirements, the AUC seems to be the best choice for evaluating models of fixation selection.

#### The effect of pooling over subjects

**Figure 1:**
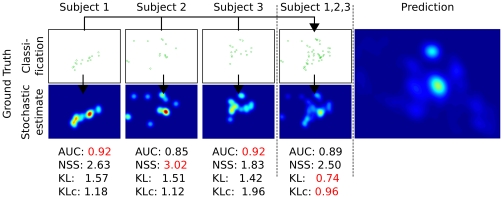
Predicting the joint fixation selection process of several subjects vs. predicting individual subjects. The prediction in this case was generated not from a model but from the fixations of several independent subjects. It therefore captures the joint process of a group of subjects. When treated as a classification problem (top row), only the fixation locations are important. In this case, the mean of the AUC or NSS scores for the individual evaluations are identical to the AUC or NSS score of evaluating the joint process. When treated as a stochastic process (bottom row; see sec:mam: sec:fdm for computational details of fixation density map estimation), locations that were fixated by one but not all subjects are less important to predict. KL-divergence, which evaluates not individual fixations but the prediction of the stochastic process, yields a better score for the evaluation of the joint process. This also holds true when it is corrected for the number of fixations in the data (KLc).

The selection of an appropriate measure is only one aspect of the evaluation process. Additionally, properties of the data against which the model is evaluated are of importance. Usually, when devising models of fixation selection , we are interested in the combined viewing behavior of several subjects, i.e. fixation data is pooled across subjects. The model should preferably predict those locations that are fixated by many subjects, because these fixations are most likely caused by salience or other factors that are stable across subjects, and not causes of fixations that are irrelevant to understanding information selection mechanisms. As a consequence of this, models that are trained to predict the joint-subject viewing behavior should perform better in predicting fixations from a set of subjects than in predicting the individual subjects from that set. This important property of model quality is not captured by the AUC and NSS. Figure 1 shows an example, where the quality of prediction as measured by AUC or NNS for the combined smooth fixation density map is just as good as the average quality of prediction of the individual subjects. That this is a general property of the NSS measure is easy to see: it takes the mean salience values at fixated locations, and for the mean it does not make a difference whether we take it for subsets individually and then average over the resulting value, or take the mean of the complete set directly. The linearity of AUC under decomposition of positive observations into subsets is less obvious, but proven in Materials and Methods: Proof of AUC linearity. In contrast, KL-divergence and correlation yield better values for predicting the joint viewing behavior, because they operate on fixation density map estimates, which take the spatial relation between fixations into account, and they are thus able to give non-linearly more weight to those locations that have been looked at by many subjects (see Figure 1). This non-linear weighting can be a good reason to consider the KL-divergence or correlation for model evaluation, despite their computational difficulties mentioned above. Deciding which of the two measures to use when one wants to exploit the effect of pooling over subjects is a difficult questions. Both measures are not robust, and both have the potentially disadvantageous property of being sensitive to non-linear monotonic transformations of the prediction. Correlation has the advantages of boundedness and being slightly less sensitive to some rescalings of the model output. However, the intuitive interpretation of its scale breaks down and becomes misleading if the dependence that is being measured is not really linear. KL-divergence is extremely sensitive to low (close to zero) predictions for locations that get a higher empirical salience, but is conceptually more appropriate for comparing probability distributions. In the end, both measures are not optimal, but because of its sound theoretical basis, we recommend using the KL-divergence when one wants to capture the ability of the model to exploit similarities in the viewing behavior within a group of subjects. In practical applications of this measure , one should also be aware of an additional complication: KL-divergences are dependent on the number of fixations used to compute the fixation density maps (sec∶mam: sec∶fdm). As a result, values which are estimated from different numbers of fixations are not directly comparable. For example, when the average fixation duration in an experiment with fixed viewing time per stimulus is dependent on image category, this can confound a comparison between categories. In Materials and Methods: Correction of KL divergence for small samples we investigate this dependency and describe a method for correcting KL-divergence scores for the bias introduced by limited data by exploiting the measure's relation to information entropy. In summary, the linearity of AUC under decomposition into subsets and the sensitivity of KL-divergence and correlation for joint-viewing versus single-subject behavior are both relevant whenever a model of fixation selection is evaluated against fixation data. KL-divergence is especially appropriate when fixation data from a group of subjects are the target of a prediction.

#### Intermediate summary

This section focused on a theoretical investigation of different evaluation measures that are used to evaluate models of fixation selection. We conclude that AUC excels with respect to our list of desired properties: The disadvantage of non-intuitive interpretation of the meaning of the AUC is outweighed by it's non-parametric nature, boundedness, robustness and compatibility with small sample sizes. In practice, it is often useful to average evaluation scores across subjects and images in order to reduce the variance introduced by small sample sizes. The linearity of the AUC ensures that these averages retain a meaningful interpretation. This property, however, comes at a cost. When the goal is to predict consistent fixation behavior across all subjects, more weight should be given to locations that are consistent between observers. Here we recommend the use of the KL-divergence. However, it is important to employ algorithms that minimize a systematic bias in the case of few data points available (see Materials and Methods: Correction of KL divergence for small samples).

### Properties of fixation data

**Figure 2:**
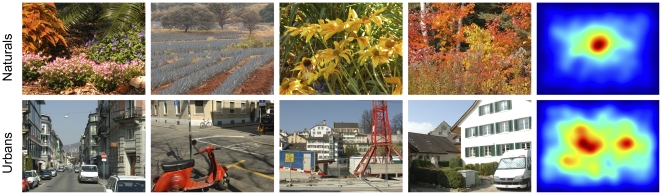
Four representative exemplary stimuli from each category used in the eye-tracking study. The top row shows natural scenes, the bottom row shows examples from the urban scenes. The right-most panels depict the spatial distribution of the first 15 fixations across all 64 images and 48 subjects in the two categories. On the natural scenes, there is a rather strong central fixation bias, while on the urban images fixations are more spread out.

The aim of the second part of this work is to investigate the upper and lower bounds on the prediction performance of fixation selection models. To this end, we examine the image and subject independent spatial bias on the one hand, and image-specific inter-subject consistency on the other hand. We use data from an eye tracking study carried out previously in our group (see sec∶mam: sec∶experiment for details and Figure 2 for some examples of stimuli). We first analyze what kind of predictions can be achieved purely from the spatial bias without any knowledge of the image that is being viewed, and evaluate how this lower bound is influenced by the number of subjects and images available for its estimation. Secondly, we describe a method for computing an upper bound for model performance that is based on ‘inter-subject consistency’ and investigate in how far it depends on the number of subjects used for its computation.

The upper and lower bounds are based on predictions blind to the predicted subject. Notably, the inter-subject consistency ignores subject idiosyncrasies. The question thus arises whether the upper bound proposed here is really an absolute upper bound for the predictive power of models of fixation selection . We therefore investigate firstly whether knowledge of the subject idiosyncrasies can be utilized to improve predictions, and secondly whether we can combine image- and subject-specific information to surpass the upper bound given by the inter-subject consistency.

#### Estimating the lower bound for fixation selection models

A way to estimate the lower bound for performance of fixation selection models is to compute the predictive power of the spatial bias. This prediction does not exploit information specific to the image or subject whose fixations are being predicted. Thus it has to be surpassed by any valuable model of fixation selection . Here, we take into account that the spatial bias varies between different image classes (Figure 2). We estimate the lower bound for NSS and AUC as the best representatives of central tendency measures and classification measures. As the results for AUC and NSS are qualitatively very similar, only the former is further considered here. More details on NSS results can be found as reference values in sec∶mam: sec∶references. Since we explicitly wish to consider small data sets, KL divergence is not suitable here (but see sec∶mam: sec∶references). To obtain a better understanding of the reliability of the lower bound, we investigate the dependence of the estimation quality on the number of subjects and images used. Specifically, we compute the lower bound by predicting fixation patterns of one subject on one image (the test set) with fixation data from other subjects on other images (the training set). To predict fixations in the test set, we construct an FDM from the training set and interpret it as a prediction for fixations in the test set. To quantify the quality of this prediction, we compute the AUC and NSS between the calculated FDM and fixations in the test set. To assess the dependence of the spatial bias estimation quality on data set size, we vary the number of images and subjects used to create the FDM. In detail, we individually increase the number of subjects and images in the training set exponentially from 1 to the maximum in seven steps (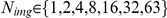; 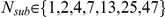). For each of the 49 combinations, we use every image and subject combination as the test set 47 times such that each of the repetitions is one random sample of images and subjects for the training set. To avoid using specific subject-image combinations more often than others, we treat cases in which we draw only one or two images or subjects separately. In this case the training set is explicitly balanced over repetitions and different test sets. In the other cases the large number of possible combinations ensures a roughly even sampling. We report the predictive power of the spatial bias as the mean over test subjects, test images and repetition.

**Figure 3:**
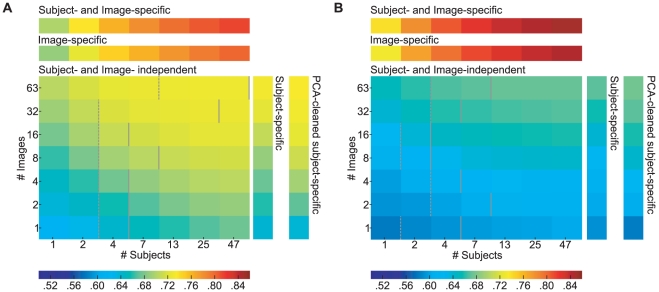
Estimation of lower and upper bounds for natural (A) and urban scenes (B). All data shown are AUC values averaged over all predictions of single subjects on single images in a given parameter combination. The predictions are based on a spatial bias (large matrix, ‘Subject and Image independent’), a subject-specific bias (column next to the matrix, ‘Subject-specific’), a PCA-cleaned subject-specific bias (rightmost column), an image-specific bias (row above the matrix, ‘Image-specific’, also referred to as inter-subject consistency) and the combination of image and subject-specific bias (topmost row). The ‘Subject and Image independent’ scores depend on the number of subjects and images used for the prediction and represent a lower bound for fixation selection models. The ‘Image-specific’ scores also depend on the number of images and yield an upper bound for fixation selection models. Comparing ‘Subject-specific’ and Subject and Image independent reveals the effect of using a subject-specific bias. The dashed lines indicate at what subject group size the subject-specific bias stops being significantly better than the spatial bias (paired t-test, 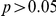). The subject-specific bias is not significantly different from the spatial bias between the dashed and solid lines. See main text for more detailed descriptions.

The spatial bias depends on the image category (Figure 3, naturals and urban scenes left and right respectively, 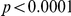). Furthermore, an increasing number of subjects (Figure 3, rows of large matrix, 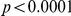) and images (Figure 3, columns of large matrix, 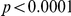) significantly increase the predictive power of the spatial bias estimate (three factorial ANOVA, category X number of subjects X number of images). For natural scenes (left) the increase is steeper than for urban scenes (right) and thereby suggests that eye-movement patterns across subjects and stimuli are more similar during the viewing of natural scenes. The predictive power of the spatial bias estimate reached for the maximum number of subjects is surprisingly high (AUC of 0.729, 0.673 for naturals and urban scenes respectively) and poses a challenging lower bound for prediction performance. The predictive power of the spatial bias estimate increases extremely slowly when more than 32 images and 25 subjects are used, implying that the estimation becomes reliable at this point. A smaller number of subjects can be compensated by a larger image set and vice versa. However, using too few data leads to a danger of underestimating the lower bound and thereby overestimating one's model quality. In conclusion, the reliability of the lower bound estimation depends on the size of the data set; for all practical purposes, 32 images and 25 subjects seem to be sufficient for a reliable estimate.

#### Estimating the upper bound for fixation selection models

To derive the upper bound for fixation selection models, we estimate the inter-subject consistency analogously to the spatial bias reliability. The rationale is that, due to variance across subjects, models that do not account for individual idiosyncrasies cannot perform perfectly. Therefore, comparing model scores to a score obtained by predicting fixations from one subject with other subjects provides an intuitive normalization. If the model score and inter-subject consistency are equal, the model predicts a new subject's fixations as well as other subjects' fixations would. In the following, we investigate the dependence of inter-subject consistency on the number of subjects used for the prediction. To estimate inter-subject consistency, we first separate subjects into a test and a training set and compute an FDM from the training set. Then, we measure how well this FDM predicts the one subject in the test set. In contrast to above, the images in test and training sets are identical. To obtain a maximally accurate estimate of the training set size for which inter-subject consistency saturates, the number of subjects in the training set is increased in steps of one. Similar to the procedure above, we use every subject and image combination 47 times as test set for every possible number of subjects in the training set. For each of the 47 repetitions a random set of training subjects is drawn. The cases where only one or two training subjects are drawn are explicitly balanced across test subjects. In the following, we report the mean AUC over test subjects, test images and repetitions as a measure of inter-subject consistency. As expected, the inter-subject consistency increases with the number of subjects in the training set (Figure 3, second row from top ‘image-specific’ in panels A and B, 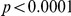; one factorial ANOVA with number of subjects as factor; additional datapoints omitted for clarity). With the maximum number of training subjects, AUC is 0.802 for naturals and 0.846 for urbans. In contrast to the pure spatial bias predictions, predictability is higher for urbans than for naturals. This results in a dynamic range of the AUC between lower and upper bound of 0.073 and 0.173 for naturals and urbans respectively. Looking at the development of inter-subject consistency with increasing subject set size, it is reasonable to assume that further increasing the training set would not have a strong effect. The second derivative of the curve is always negative, suggesting that the curve saturates. For example, from 20 to 21 subjects, the increase is 0.001, from 40 to 41 it is only 0.0002. Thus, for all practical purposes, the inter-subject consistency of about 20 subjects constitutes an upper bound for generic models of fixation selection in free viewing tasks.

#### Subject-specific spatial bias

To investigate the importance of subject idiosyncrasies for the prediction of fixation locations , we examine whether knowledge of a subject-specific spatial bias is more valuable than knowledge of the bias of other subjects. To that end, we estimate how well a subject-specific spatial bias predicts fixations of the same subject on other images. We proceed as before and predict fixations in the test set with an FDM based on fixations in the training set. For every combination of the number of predicting images, test subject, and test image, we use 63 different training sets. The images in the different training sets are randomly sampled and the subject is the same in training and test set. The random samples are balanced explicitly if there are only one or two images in the training set. Analogous to the generic spatial bias, the subject-specific spatial bias's predictive power is dependent on the number of images used for estimation (Figure 3; vertical bar ‘subject-specific’ directly to the right of the large matrix in panel A and B, 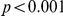, ANOVA with number of images as the only factor). For any number of images, the subject-specific spatial bias is more predictive than the predictive power of a single independent subject (Figure 3 compare left-most column in the central square to vertical column directly to the right). However, it is not higher than the predictive power of the best spatial bias, obtained from a set of 47 independent subjects (Figure 3 compare right-most column in the central square to vertical column directly to the right). With the exception of 63 images from the ‘natural’ category, the bias from a large number of subjects achieves better performance than the subject-specific bias. The exact number of subjects that is needed to achieve better performance than the subject-specific spatial bias depends on the number of images (see dashed lines in Figure 3). The improvement in AUC over a generic prediction based on a single independent subject ranges from 0.009 on urbans and 0.021 on naturals for a single image to 0.017 on urbans and 0.029 on naturals for 63 images. The increase in predictive power of the spatial bias achieved through incorporating subject-specific information might appear small, but it is a sizable fraction of the dynamic range between lower and upper limit (0.073 and 0.173 naturals and urbans respectively), and significant for all numbers of training images (paired T-tests over 48 subjects, 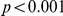).

#### Combining the positive effects of knowing the correct subject and knowing many subjects

**Figure 4:**
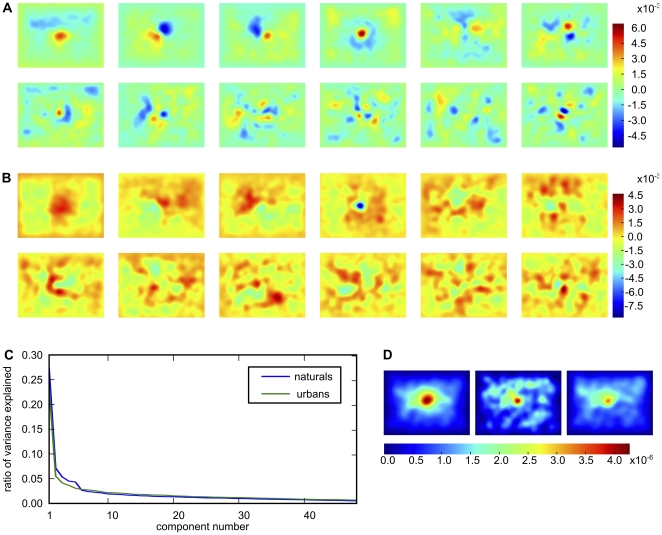
PCA-based cleaning of a subject-specific spatial bias. Panels A and B show the first 12 principal components respectively for naturals and urbans. For demonstration purposes, the underlying subject biases were computed with fixation data from all images and all subjects. Please note that the sign of the principal components is arbitrary. Panel C shows that the variance explained by each component drops dramatically. This, and the fact that the first 5 components carry some interpretable meaning, led us to choose the first five components for the cleaning of the subject-specific bias. Panel D shows an example of this. The left plot shows the spatial bias of all other subjects, the center one the subject-specific bias and the right plot shows the result of reconstructing the subject-specific bias with the first five principal components.

We have seen that the prediction of the spatial bias from one independent subject can be improved on in two ways. By incorporating information from more independent subjects ((see Estimating the lower bound for fixation selection models), reducing the uncertainty in the estimation of the true spatial bias, or by using subject-specific information ((see Subject-specific spatial bias). Both improvements have effects of similar sizes. It seems possible that combining both methods would allow an even better prediction. We hypothesize that the spatial bias of a large set of subjects consists of certain identifiable components, to which individual subjects contribute with different strengths. In that case, it should be possible to express an individual subject's spatial bias as a combination of these components. Such an approach would be more reliable, because the components can be estimated from many different subjects, effectively reducing the noise in the estimate. To identify these components, we compute the spatial bias for all training subjects on a given number of images, and perform a principal components analysis (PCA) on these biases. Figure 4 A,B shows the first 12 principal components of an exemplary case, which are the directions where the spatial bias varies most over subjects. Importantly, the amount of variance explained by the components drops rapidly (see Figure 4C). Hence the first few components explain the larger part of variance of the data and the remainder is increasingly noisy and uninformative. To enhance the reliability of the estimate, we only keep the first 5 components. We incorporate subject-specific traits by finding subject-individual weights for the components. These weights are computed by regressing the components onto the subject-specific bias, which is computed on all images in the training set. Figure 4D illustrates the subject-specific weighting of the multi-subject spatial bias. This combines the subject specific information and the statistical reliability of a large data base.

Importantly, we do not use the subject or image to be predicted for estimating the components. To evaluate the efficacy of this approach, we carry out the same subject evaluation as for the evaluation of the sec∶subsb, but use the described PCA method instead of the regular individual subject bias. This procedure combines two possible sources of improvements: subject-specific information and noise reduction in the spatial bias estimate. To ensure that the subject-specific weighting of principal components has a separate effect, we also evaluate how the PCA spatial bias cleaning without subject-specific weighting performs. For this control, we simply weight the first five components with their eigenvalues and use their sum as the prediction. In order to evaluate whether this method is able to combine the positive effects of knowing a specific subject and of having a robust estimate from many subjects, we need to compare it to both individual methods.

**Figure 5:**
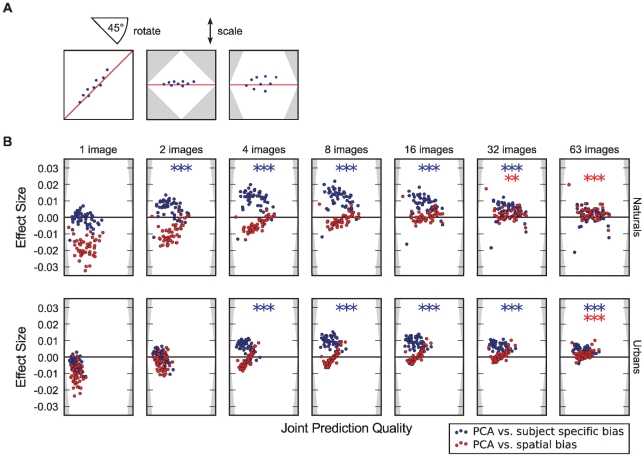
The effect of using a subject-specific PCA cleaned bias for prediction. Panel A explains how the plots in B come about. We scatter the AUC score for predicting individual subjects averaged over images and repetitions with the PCA-cleaned bias against either the scores for the subject-specific or average spatial bias. For better visibility we rotate the plot by 45 degrees and sclae it. This causes the x-axis to become a measure of how well a subject can be predicted with either method and the y-axis becomes a measure of effect size, i.e. how much the prediction improves by application of the PCA. Please note, the y-axis is labeled such that it indicates the difference between the two scores and not the distance to the diagonal. To make the effects more visible we scale the y-axis to include the relevant range. The blue dots compare the effect of using PCA-cleaning to the subject-specific bias. It can be seen that in both categories the effect of the PCA depends on the number of images. The asterisks indicate that the effect size is significantly larger than zero (paired t-test, *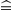 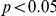, **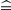 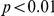, ***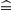 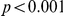).

First we investigate the improvement in predictive power in comparison to the subject specific spatial bias (Figure 5). In case a single natural image is used to compute the principal components no improvement is observed. For an intermediate number of images a significant improvement (paired t-test, 48 subjects, significance level indicated by number of asterisks) compared to the subject specific spatial bias is demonstrated (Figure 5B upper row, significant deviation of blue dots from the horizontal axis that was the main diagonal in the original scatter plot). Testing subjects on even larger numbers of natural images leads to a smooth distribution of the spatial bias and no further improvement by PCA-cleaning is achieved. In the case of urban images an improvement is observed in a range from 4 to 63 images (Figure 5B lower row), which is shifted by a factor of two compared to naturals. Hence, in comparison to the subject specific spatial bias PCA-cleaning boosts performance by a modest degree for the case of testing with an intermediate number of images. Second, we compare prediction performance of PCA-cleaned individual spatial bias to the average obtained by a large number of subjects. Here we observe a small but significant improvement only for a larger number of images (Figure 5B significant deviation of red dots from the horizontal axis). The small effect size might be expected because there is already so little noise in the spatial bias for one subject. Thus, the predictive power of the generic spatial bias is already very high, leaving little room for improvement. On the other hand, the results for a small number of images illustrate that the PCA cleaning requires a certain amount of data to work properly. There is a possibility that the subject specific weights do not contribute to the observed effect, but that PCA-cleaning is only effective by removing noisy components. To control for this we repeated the same analysis but omitted the subject-specific weighting and instead weighted the components with their eigenvalues obtained from the PCA. This does not lead to a change in predictive power compared to the pure spatial bias (paired T-test, 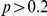; data not shown). In summary, the PCA cleaned subject-specific spatial bias estimate combines the positive effects of reliable bias estimation and exploiting subject-specific traits.

#### Predicting better than perfect: combining subject- and image-specific biases

**Figure 6:**
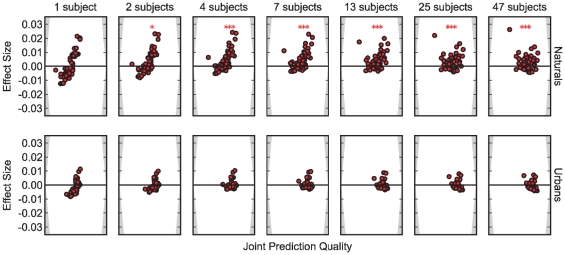
Combining a subject-specific and image-specific spatial bias for a better than perfect prediction. The plots are produced as in Figure 5. The effect depends on the number of subjects that enter the bias estimation and the image category. For natural scenes, a statically significant effect (paired t-test, *** = 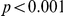) can be seen when four subjects or more are used. The effect cannot be seen for urban scenes, which might be explained by the low predictive power of the subject-specific bias compared to the high predictive power of the image-specific bias on urbans.

The previous section showed that subject-specific predictions can improve the already good prediction of a large group of subjects in the domain of the spatial bias. After estimating the upper bound for fixation selection models, we established that the inter-subject consistency marks an upper bound for prediction quality of subject independent models. Given these observations, the question arises whether subject-specific models can surpass the inter-subject consistency bound. As a proof of concept, we combine inter-subject predictions with the subject-specific spatial bias as a simple form of subject-specific information, and analyze if this procedure can lead to a better prediction. We assume that viewing behavior on an image is driven partly by a subject-specific spatial bias and by image properties, i.e. the inter-subject prediction contains both components. The idea is to replace the general spatial bias in the inter-subject fixation density map with a subject-specific spatial bias while keeping the image dependent part. To achieve this, we first compute the fixation density map of all training subjects on the image in question, i.e. the inter-subject prediction. Second, we remove the general spatial bias by dividing the inter-subject prediction point-wise through the training subjects' spatial bias computed on all other images. To arrive at a prediction, we multiply the resulting image-specific bias point-wise with the spatial bias of the predicted subject. Finally, we normalize the resulting map to unit mass and evaluate how well it predicts the fixations of our test subject. We use the same cross-validation procedure as for the generic inter-subject predictions, but limit the computations to the logarithmically increasing training set sizes used for the spatial bias evaluation. Inter-subject consistency is recomputed for these new training sets to allow paired tests between subject-specific and generic predictions. The results show a small but significant effect on naturals (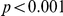, paired t-test for 4 or more subjects. See Figure 6). For example, the improvement for 47 subjects is a mean AUC increase from 0.809 to 0.815. There is no significant effect on urbans (paired t-test, 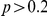 for all numbers of subjects). The difference between categories can probably be explained by the fact that the spatial bias has less predictive power for urbans and that the inter-subject consistency is already higher in urbans. We conclude that the combination of subject-specific information and image-specific information can surpass the inter-subject consistency upper bound on natural but not on urban images.

We draw five different conclusions: First, the lower bound, based on the image- and subject-independent spatial bias, is surprisingly high (AUC of 0.729 and 0.673 for naturals and urbans respectively) but the reliability of the estimated bound depends on the size of the data set. For all practical purposes, 32 images and 25 subjects seem to be sufficient for a reliable estimate. Second, the reliability of the upper bound, which is based on the consistency of viewing behavior between subjects, also depends on the data set size. For all practical purposes, the inter-subject consistency of about 20 subjects is sufficient to establish an upper bound for generic models of fixation selection in free viewing tasks. Third, the incorporation of subject-specific information can significantly improve the predictive power of the subject- and image-independent spatial bias. Fourth, the predictive power of the spatial bias can further increase when the subject-specific information is de-noised with information from other subjects. Fifth, the dependence of the upper bound on joint-subject processes makes it possible to surpass this bound by combining subject- and image-specific biases.

## Discussion

In this work, we have focused on how models of fixation selection can be evaluated. Based on theoretical considerations, we argued that the AUC is the best choice for the kind of data that is usually available in eye-tracking studies. However, when predicting viewing behavior that is consistent across a group of subjects, KL-divergence presents itself as a superior alternative, given that the data set is large enough. Regardless of the measure, model evaluation is also influenced by the inherent properties of eye-tracking data. In particular, the predictive power of the pure spatial bias estimate poses a challenging lower bound for prediction performance that any useful model has to exceed. Moreover, the inter-subject consistency constitutes an upper bound for generic models of fixation selection . The accuracy of the estimate for both bounds depends decisively on data set size.

By using these bounds as a reference frame, we showed that subject idiosyncrasies can be exploited to increase the prediction performance. This can be pushed to the point where the predictive power surpasses the inter-subject consistency bound. From a more general perspective, the two bounds discussed in this paper form a reference frame that allows for a substantially more informed assessment of the quality of a model of fixation selection than just a measure score alone. It is essential that these bounds are reliably estimated by acquiring enough data. To see this, consider a case in which data is only available from a small set of 10 subjects. In this case the inter-subject AUC and the predictive power of the spatial bias will be underestimated. Both these effects subsequently lead to an overestimation of model quality. The following two examples illustrate the advantages of our approach when this caveat is kept in mind.

First, if we consider a task that induces a very specific spatial bias (e.g. pedestrian search, [14]), the AUC score depends on how much of the image is covered by the task-relevant area. People will look for pedestrians on the ground, so in principle it is possible to increase the area of the sky, e.g. by decreasing the camera's focal length, without substantially changing fixations patterns. If our model has also learned to ignore that additional spatial region, the AUC is increased substantially. Yet we would not claim that the increased AUC reflects a better description of the fixation selection process. Reporting the predictive power of the pure spatial bias alongside the model's score allows a fair evaluation of a model in all cases.

Secondly, in our data we found that the category where the spatial bias is weaker (urbans) has a stronger inter-subject consistency. This double-dissociation has important consequences for the evaluation of fixation selection models. One and the same model, incorporating both spatial bias and image statistics, may score higher on naturals than on urbans, because of the predictive power of the spatial bias. On the other hand, if a model is almost optimal and comes close to the predictive power of other subjects' fixations, it will score higher on urbans. Thus, the type of dataset the model is evaluated on will have an effect on one's judgment of model quality. As a result of this, a comparison of different models is nearly impossible if they were evaluated on different data sets, unless the upper and lower bounds for the specific datasets are explicitly given.

A different, commonly used method to control for the spatial bias when using AUC is to sample the negative observations not from the whole image, but only from points that have been fixated on other images [10]. If this is accompanied by an equally corrected report of inter-subject consistency, it allows for an unbiased model comparison much in the same way as reporting upper and lower bounds as proposed here. In the context of model evaluation, however, we believe that explicit is better than implicit, i.e. that reporting the complete reference frame gives the reader a more direct grasp of the model's capabilities. We conclude that the most comprehensive way to evaluate a model of fixation selection, especially with respect to comparisons between different models, is to use AUC and/or KL-divergence as performance measures, and to report both the predictive power of the spatial bias and the inter-subject consistency of the data set that the model is tested on.

Besides putting model performance into perspective, the proposed reference frame can also be of use prior to model evaluation. The two bounds define the dynamic range for predictions of the distribution of fixation points. The ideal data set for evaluating a model of fixation selection would have a large range, indicating that subjects fixate different locations on different images - limiting the predictive power of the spatial bias - but agree on the selection of fixation points on single images. When the predictive power of the spatial bias is small, models of fixation selection can only improve by uncovering regularities distinct from the spatial bias. At the same time, high inter-subject consistency indicates that a common process regulates the selection of fixations in observers, and it is this process that models of fixation selection target.

With a change in perspective, the reference frame can be used to probe for differences in viewing behavior. The lower bound indicates to what extent subjects' viewing behavior is independent of the image, whereas the upper bound quantifies their agreement. This not only allows interesting comparisons between different groups of subjects, but also provides a tool to investigate the effect of different stimulus categories. In this work, we investigated urban and natural images and found that the range of the reference frame is larger on urban than on natural images. This shows that urban images elicit higher subject agreement in fixation selection and evoke a stronger image-dependent component in fixation target selection. The cause of the differences between categories is an interesting topic for further investigation.

**Figure 7:**
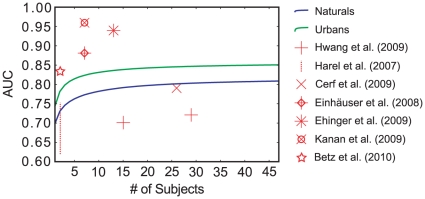
A comparison of inter-subject consistency AUC in different studies. Green and blue lines show the dependence of inter subject consistency on the number of subjects in our data. The symbols show inter-subject consistency values reported in other studies. All studies that reported higher values used either stimuli that contained a wealth of high-level information or employed a specific task. Cerf et al. [12] also use a free viewing task and are compatible with our findings. Harel et al. [17] only report a range of values (read from a figure). Notably, Hwang et al. [13] use image rotations to diminish top-down influences and observe lower inter-subject consistency.

The inter-subject consistency has been used before as an upper bound for model performance, which allows for a direct comparison of our values and the ones provided in the literature. Interestingly, we found that on first sight not all values were in line with our results (Figure 7). However, there seems to be a consistent explanation for the deviations: All values of inter-subject consistency that lie above those found in our data were computed on data where there was an explicit task during the eye-tracking experiment (object naming [16] or pedestrian search [3], [15]), or the stimuli material contained a wealth of high-level information (web-pages [21]). On the other hand, Hwang et al. [13] explicitly designed their experiment to minimize high-level information by rotating the images by 90 or 180. They report lower inter-subject consistency, but the effect of group size is in line with our results. Finally, Cerf et al. [12] use a free viewing task similar to our experiment and obtain values almost identical to ours. We conjecture that inter-subject consistency is strongly influenced by the subjects' task and the availability of high-level information. This is also in line with the category differences found in our data (urbans 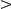 naturals), since the urban scenes provide more high-level information (e.g. man-made objects, people), as well as with category differences reported by Frey et al. [30]. Interestingly, high inter-observer consistency is not related to a large influence of the spatial bias. In our dataset, the former is higher for urban scenes while the latter is higher on natural images. A speculative explanation of this finding is that when high-level information is present in an image, it will guide the eye movements of many subjects to locations that are not necessarily in the center of the image, increasing inter-subject consistency and decreasing the influence of the spatial bias. In the absence of high-level information, subjects tend to look more towards the center of the screen, but in a less homogenous fashion. This fallback strategy leads to an increased spatial bias and decreased inter-subject consistency. Further evidence for this hypothesis comes from eye-tracking studies with pink-noise stimuli, which are completely devoid of high-level information and where the influence of the spatial bias is comparatively large [31]. Our analyses of subject idiosyncrasies relative to our established bounds showed that the increase in performance, although statistically significant, is very small. In the case where data from 63 images are used, knowing the spatial bias of a specific subject is as good as knowing more than 7 other subjects on naturals, or knowing more than 2 other subjects on urbans. The smaller effect for urbans fits the observation that inter-subject consistency is higher in that category, making knowledge about a specific subject less unique. This relates to a possible reason for the small overall effect size in both categories: Acik et al. [31] show that different demographic subject groups have remarkably different viewing behavior. Specifically, explorativeness, a property that is closely related to the spatial bias, decreases with increasing age. Our subject group consisted exclusively of university students between 19 and 28 years of age. Thus it can be expected that the effect of knowing the subject to be predicted would be much larger in a more heterogeneous subject group with lower inter-subject consistency. In such a scenario, the improvement caused by PCA-cleaning demonstrated in the present study could become more relevant. In general, the PCA-cleaning requires fixation data on a fair number of images for a good signal to noise ratio. In practice, the principal components could be determined from a large set of subjects and images recorded in a baseline study. It may then be possible to tailor a clean subject-specific spatial bias based on fixations from the subject of interest on few images. This technique may be useful in a modeling context, when the goal is to fine-tune a generic model for predicting individual subjects' fixations.

The spatial bias is of course only one feature of viewing behavior where subject idiosyncrasies can play a role. There are possibly many different ways to incorporate these into a model of fixation selection. An obvious candidate would be the relative importance of different image features in a bottom-up model. Whether subject-specific modeling of feature weights has a positive effect is an interesting question for further research, but goes beyond the scope of this article.

Finally, we showed that it is possible to surpass the limit set by the inter-subject consistency when incorporating subject and image-specific information into the prediction. Despite the very small effect, this result exemplifies the potential value of subject-specific predictions. However, it also reveals another aspect of the evaluation of models of fixation selection. Judging only by the AUC values, we have created a prediction that exceeds the inter-subject consistency bound and incidentally also the best prediction ever described in the literature. In a sense, our prediction is better than what has previously been called ‘perfect’. Of course no sensible person would congratulate us on this achievement. Rather, it shows that claims about theories of fixation selection based purely on a prediction's AUC values, or the percentage of inter-subject AUC achieved, can be quite hollow.

A decisive question that should be part of every model evaluation is what we can learn from this model about processes of fixation selection implemented in the brain. Good models do not only achieve high prediction scores, but also reproduce and, better, explain differences in human viewing behavior, such as the different reference frames between natural and urban images, or the temporal evolution of scan paths. Models that replicate novel aspects of viewing behavior might still be revealing about the underlying mechanisms, despite having low predictive power. Here, we have to consider two questions: do we understand the mechanism by which our model goes from input to prediction? And is this mechanism plausible? If we can answer both these questions in the affirmative, and our model performs well on an adequate stimulus set under the evaluation procedures described in this article, we really will have made a contribution.

## Materials and Methods

### Theoretical maximum value for AUC

In the present work, receiver-operating characterisics (ROC) ((see Existing measures) analysis is applied to classify fixated locations vs. non-fixated locations. This treats the prediction of fixations as a discrete binary problem: a location is either fixated or it is not. However, for an unbounded number of subjects and taking into account finite precision of the occulomotor system and the eye-tracker, there is no principled reason why a location cannot be fixated and therefore all locations should eventually be fixated. This implies that every location has a finite probability to be selected as fixated and a finite probability to be selected as non-fixated. Hence, classification of a location inherently carries an error, as it is neither perfectly fixated nor non-fixated. It follows that an AUC of 1 is not achievable and a bound lower than 1 does exist.

In this section, we formalize these considerations and derive a quantitative estimate of the upper bound of the area under the ROC curve when we conceptualize the prediction as a probability density function. In the following we redefine the hit and false alarm rate for calculating the AUC value to work with probability distributions. The observed distribution of fixation points upon presentation of stimulus 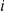 is described by 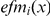, with 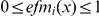 for all 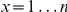. The 2D topology is irrelevant, as there is no interaction between different positions, hence we can use a one dimensional index. Furthermore 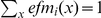. We assume that for all 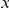 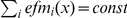. This means that for every location, across all images, the probability of fixations is constant, i.e. there is no spatial bias. A spatial bias leads to additional complications like equilibrating the spatial discretization to achieve a constant distribution of control (non-fixated) locations. It does, however, not change the principle result. We furthermore assume that the prediction of fixated regions 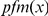 is perfect when 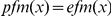. Now we evaluate the quality of this prediction in terms of ROC. For a threshold 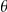 the number of hits is given by
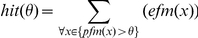



We classify as a fixated all locations where the prediction exceeds the threshold, and weight each such location with the empirical probability that this point is fixated. Above we assumed 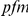 equals 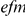 and we simplify
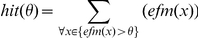



Because of all 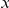 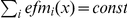 and 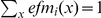 the distribution of control fixations is flat at a value of 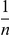 and the number of false alarms is
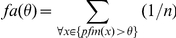



Again we count all locations where the prediction exceeds the threshold, but now weight each such location with 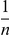. As before, the predicted map equals the empirical one and we have
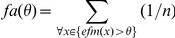



For any non-degenerate distribution where 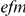 takes on values other than 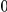 and 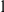 there must be a threshold where 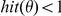 and 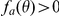. Hence the area under the ROC curve is smaller than 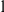.

What is the upper boundary of the AUC for a specific 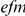
? Given
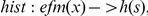
with 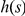 the frequency of occurrence of a specific saliency value 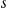. 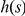 has some important properties:
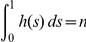
the spatial discretization of 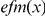 is 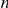 and because 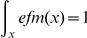 also
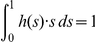
is a probability density distribution with integral 1. For a given 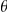 the false alarm rate is given by
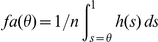



The integral yields the number of points above the threshold which is weighted with 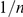. The hits are given by
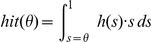



When using these definitions of hits and false alarms the AUC is given by
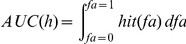



Note that the false alarm rate increases as we lower the threshold from 1 downward. By change of variables we obtain
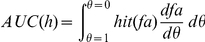
changing the bounds
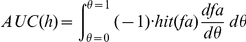



As 
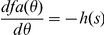
 (see definition of 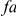 above) we obtain
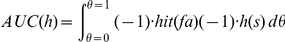


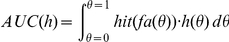


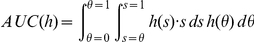



This formula yields the upper bound for predicting a given empirical fixation map.

### Proof of AUC linearity

Here, we prove that the value of the area under the receiver-operating characteristics curve (AUC) for a given multiset of positive (P) and negative (N) observations does not depend on how the positive observations are grouped, i.e.
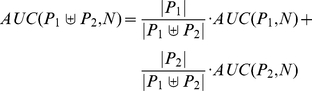
(1)where 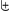 denotes the multiset union. As a given location may be fixated several times the notion of a multiset seems appropriate. Multisets are a generalization of sets and may contain multiple memberships of one and the same element. The AUC is obtained through trapezoidal approximation of the area under the curve plotting the true positive rate (TPR) against the false positive rate (FPR) for all thresholds, according to:
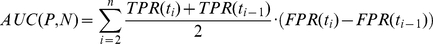
(2)

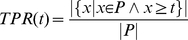
(3)

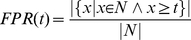
(4)

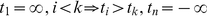
(5)


#### Lemma

Let 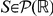 be a finite set of real numbers and 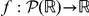 be a function, such that for each 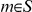 hold
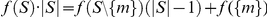



That implies for any set 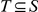


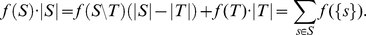



This can easily be seen through induction over 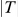, beginning by 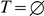


The Lemma reduces (1) to
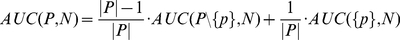
(6)


From (3) follows
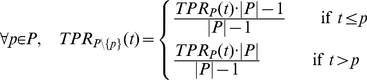
(7)


Now we can compute 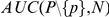 and 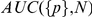. Let 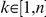 be the smallest value for which 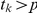, then
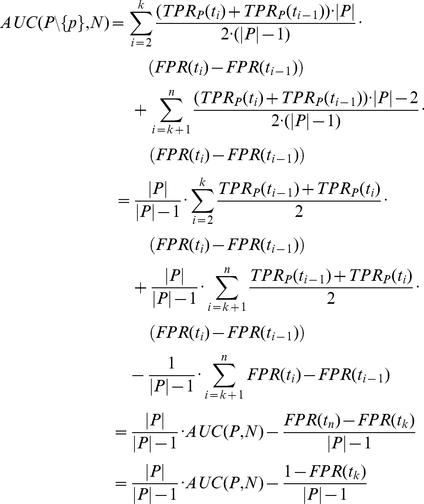
(8)and
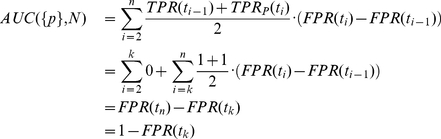
(9)


Using (8) and (9) it is easy to see that (6) is true, proving (1).

### Computational details of AUC analysis

Although in theory AUC is independent of arbitrary parameters, this is not entirely true in practice. Strictly speaking,the ROC curve plots the probability of a hit against the probability of a false alarm, and these probabilities of course have to be estimated. However, we have found that when applying this measure to the evaluation of models of fixation selection, using relative frequencies as an estimation of probabilities works well and can be seen as a sensible default value that requires no further parameters. In that case, there remain two decisions on related issues that have to be made when computing the AUC, and both influence the resulting value: first, we need to decide which thresholds to use to create the underlying ROC curve, since an infinite number of thresholds with infinitesimal spacing is not achievable. Second, it has to be decided how the area under the ROC curve is computed. In general, trapezoidal integration is the method of choice. However, in the special case of fixation classification, there is a simpler way. Here, it is usually the case that we have a very large number of negative values (either all values in the salience map, or all values that were not fixated, or all values at locations that were fixated on other images) and a smaller set of positive values (salience values at fixated locations). Obviously it suffices to use all unique values in the combined set of positives and negatives as thresholds. Neither the true positive rate nor the false positive rate will change for any other threshold values. In general, the true positive rate can only increase for threshold values in the set of positives. All other thresholds, those in the set of negatives, can only increase the false positive rate while the true positive rate remains constant. This implies that the ROC curve approaches a step function and the thresholds in the set of actuals define the steps. In a step function, there is no difference between trapezoidal integration and lower sum integration. And since the thresholds from the set of actuals define the steps, it suffices to use lower sum integration with only these values as thresholds. There is one pitfall that has to be avoided with this approach. When no threshold reaches a true positive rate of one before the false positive rate is one, the AUC can be underestimated. If this is the case, we use trapezoidal integration for the last segment of the curve. This method, which is computationally much more efficient, as it involves fewer threshold values, was adopted for all reported AUC values in this article.

### Fixation density map estimation

In the analysis of eye-tracking data, we make frequent use of fixation density maps (FDM), which estimate the probability that a specific location is fixated. These are computed by smoothing a two-dimensional histogram of fixations, where each pixel is one bin, with a Gaussian kernel of 2 FWHM, normalizing to unit mass. The rationale for smoothing is that a) the eye-tracker operates with limited resolution (calibration-error 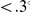) and b) the visual system samples information at high-resolution not only from a single fixated pixel but from the fovea which corresponds to about 2 of visual angle in diameter. For computational efficiency it is often necessary to scale FDMs to smaller size. This is achieved by adjusting the bin sizes of the histogram and the size of the Gaussian kernel accordingly.

### Correction of KL divergence for small samples

**Figure 8:**
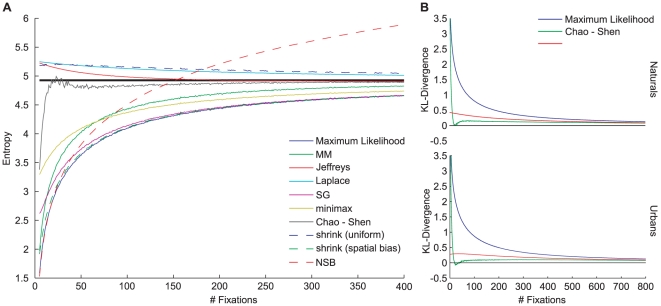
The effect of sample size on the KL-divergence. A. Performance of different methods to remove the sample size bias from entropy estimates in a simulation using eye-tracking data. The bold line shows the maximum likelihood entropy estimate computed on the entire data set (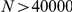) and can be interpreted as ground truth. The Chao-Shen and Jeffreys correction methods approach the target value with the lowest number of samples. Descriptions of the individual methods can be found in [35] (Chao-Shen), [32] (shrink), [36] (Laplace), [37] (Jeffreys), [33] (MM), [34] (NSB), [38] (SG), [39] (minimax). B. Sample size dependence of different KL-divergence estimation methods. The standard maximum likelihood method shows a strong positive bias for small samples, both correction methods tested can reduce this problem for sample sizes of ca. half the number of bins in the estimated distributions or larger.

The KL-divergence can be expressed in terms of Information Entropy, and for Information Entropy it is known that it systematically depends on the sample size [32]–[34]. These observations lead us to suspect that the KL-divergence is also biased, which is problematic when different models are evaluated against densities estimated from different sample sizes. We carry out two simulations to investigate the size of this potential confound. First, we treat the overall spatial bias as our prediction. We then take a random sample of fixations from the set that constitutes the spatial bias and repeatedly calculate the KL-divergence between the FDM of our sample and our prediction . If the sample gives a perfect estimate of the distribution it was drawn from the KL-divergence should be zero. We increase the number of fixations per sample from 6 to 800 in steps of 2, and draw 1000 samples of every size. Since discrete Entropy estimates are also strongly influenced by the binning of the probability density function, we do not use our standard procedure for computing fixation density maps. Instead, we sort the data into a grid of 16×12 bins (leading to N = 192). The number of grid cells was selected such that the area of each bin is equal to the area of a circle of diameter two degrees of visual angle. These FDMs are not smoothed, since they already have a coarse resolution. In a second simulation, we take a normal distribution with specified parameters (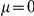, 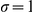) as our prediction and sample our data from a different normal distribution (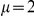, 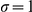). In this case the true KL-divergence can be determined analytically and the KL-divergence computed from different sample sizes can be compared to this target value. We proceed in the same way as before and increase the sample size from 6 to 800 in steps of 2 and draw 1000 samples of every size. Densities are estimated as histograms with 100 bins. In both cases the estimated KL-divergence was higher than the analytical value. The difference between mean estimated KL-value and analytical value decreased with increasing sample size (the results for simulation 1 are depicted in Figure 8A; results for simulation 2 were similar). Thus, comparing models evaluated on different data set sizes is difficult. One approach to cope with the sample size dependence of the estimate is to keep the sample size constant in every comparison by randomly sampling as many fixations from each data set as are available from the smallest one. However, if the size of a novel data set is comparably small and previous model evaluations were performed on a larger and inaccessible data set, it is not possible to reduce the larger data set. Thus, to foster comparisons between different studies, it would be advantageous to be able to directly correct for the bias introduced by sample size. There are multiple methods that try to improve the estimate of entropy values (recall that KL-divergence is directly dependent on the Entropy estimates), as compared to the typically-used maximum likelihood approach. We therefore investigate the applicability to fixation data of several methods [32]–[39], for which [32] provides an implementation. To compare the efficacy of the different approaches, we carried out simulations in which we estimated the entropy of differently sized samples from the general spatial bias. In addition to the direct relevance for the calculation of KL-divergence, an important advantage of an unbiased entropy estimate is that entropy can be used to characterize viewing behavior [31], [40], [41]. It is therefore relevant to have an unbiased estimate, e.g. for comparing different experimental conditions with different amount of fixations. The simulations follow the pattern that we used for determining the sample size dependence in KL-divergence. Due to the large number of different correction methods compared, we only draw 200 samples of each size to reduce computational load. We compare estimates for different sample sizes to the entropy of all fixations in one category (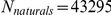,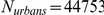), assuming that the estimate is nearly unbiased with such a large sample size. The simulations show that it is in principle possible to improve the entropy estimate. However even in the best case, the number of samples required for a reasonable estimate is approximately half the number of bins of the fixation density map. This is a large improvement over uncorrected Entropy, which requires the number of data points to be at least equal to the number of bins squared. The fixation densities in our simulations were down sampled to 169 bins. Considering that FDMs are typically smoothed with a 2deg FWHM Gaussian kernel, the effective resolution of a FDM is already much lower than the number of pixels suggests, making the down sampling tenable. Overall the correction methods proposed by Chao and Shen [35] and Jeffreys [37] work best of all tested methods. To yield a correction method for the KL-divergence, its Entropy and cross-Entropy terms have to be corrected. Starting with Chao-Shen, the pure entropy term can straightforwardly be corrected. Moreover, if we presuppose that a model output corresponds to a correct probability density (Q), we can also apply Chao-Shen to correct the cross Entropy 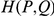. Here, we use
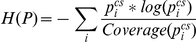


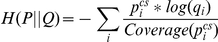
to compute the corrected KL-divergence, where pcs and Coverage are the two Chao-Shen correction terms (see [35]). The Jeffreys correction can simply be applied by adding 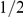 to the cell counts of the FDM before it is normalized to unit mass. To validate applicability of Chao-Shen in the case of KL, we repeated the simulations for the maximum likelihood KL-divergence estimation but used the Chao Shen and Jeffreys corrected estimation. As shown in Figure 8B, the correction substantially improves the KL estimates as compared to the maximum likelihood version. The Jeffreys correction works well on our data, which is in part due to the fact that our distribution does not deviate too much from the uniform prior assumed by the correction method. If there are strong reasons to believe that one's data deviate much from a uniform distribution, one should therefore be careful with this correction. The Chao Shen correction is very close to the true KL-divergence between the underlying distributions at a sample size of about 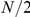.

### Description of the eye-tracking study

The study has been approved by the ethics committee of the University of Osnabrück and was conducted according to the principles expressed in the Declaration of Helsinki. All subjects gave written informed consent prior to the study and were informed of their right to withdraw at any time without negative consequences. The experiment consisted of the presentation of 255 stimuli from four different categories (naturals, urbans, fractals and pink-noise). The ‘natural’ category contains 64 stimuli that depict outdoor scenes like landscapes, forests and flowers. The 64 ‘urbans’ show rural and city scenes with many man-made structures. The images comprise a large variety of different scenes and vary over many different parameters (street scenes, buildings, differences in depth and openness, close-ups and landscape perspectives). In the urban scenes only very few persons are shown and very little text. All stimuli have a large depth of field to avoid the guidance of eye movements by the photographer. We do not use the artificial stimuli from the fractal and pink-noise categories. The task of the subjects was to freely view the pictures (‘watch the images carefully’). Each stimulus was shown for six seconds and a fixation point was shown in the center of the screen before each stimulus to perform a drift correction. The distance to the screen was set at 80 cm; the display used was a 21-inch CRT monitor (SyncMaster 1100 DF 2004, Samsung Electronics, Seoul, South Korea) with a screen resolution of 1280×960 pixels; refresh rate was 85 Hz. The stimuli had a size of approximately 28.4×21.3 degrees. 48 subjects (24 male) participated in the experiment and received either 5 or course credit as compensation. Subjects were aged between 19 and 28 years, naïve to the purpose of the study and had normal or corrected-to-normal vision. The eye-tracker used was an Eyelink II system (SR Research Ltd., Mississauga, Ontario, Canada). This head-mounted system is capable of tracking both eyes; however, only the eye giving a lower validation error after calibration was used for data analysis. Sampling rate was set at 500 Hz. Saccade detection was based on three measures: eye movement of at least 0.1, with a velocity of at least 30/sec and an acceleration of at least 8000/sec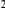. After saccade onset, minimal saccade velocity was 25/sec. The first 15 free fixations of each trial were used for data analysis. All data is available from the authors upon request.

### Reference values for spatial bias and inter-subject consistency

Here we report numeric AUC (Table 2 and 3) and NSS (Table 4 and 5) values for predicting fixations of one subject on one image with a subject and image independent spatial bias (estiamted lower bound, see Estimating the lower bound for fixation selection models) and with an image-specific bias (inter-subject consistency, estimated upper bound, see Estimating the upper bound for fixation selection models). All reported values are means across cross-validation runs, as described in Estimating the lower bound for fixation selection models. So far we omitted the computation of upper and lower KL-divergence boundaries. Testing the estimation reliability by changing the number of subjects and images in the training set would be confounded by the different numbers of fixations in the training set (our correction methods are intended for controlling the test set and thus do not apply here). To nevertheless be able to report sensible reference bounds, we restrict ourselves to a large training set size such that the influence of different amounts of fixations in the training set is small. In detail, we pick out one row (63 images, varying the number of subjects for prediction) and one column (25 subjects, varying the number of images for prediction) of the subject and image independent predictions. This leaves either many images or many subjects in the training set, such that there are at least 375 fixations in the training set. To furthermore minimize the effect of different amounts of fixations in the training set, we bin the screen into 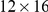 squares. The test set always contains fixations from 23 subjects, we omit the case where more than 25 subjects are in the training set, such that the number of fixations is constant at 345 fixations. The evaluation of the entropy correction methods has shown that with this amount of fixations and dimensionality of the probability density map, no correction for different amounts of fixations is needed. We also compute the inter subject consistency for predicting 23 subjects with data from the remaining 25 subjects for every image and 48*63 random assignments of subjects into test and training set. Table 6 and 7 report the mean over images and random assignments.

**Table 2:** AUC values for natural scenes.

Nr. of subjects 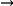 Nr. of images 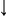	1	2	4	7	13	25	47	Subject-specific
Image-specific	0.689	0.724	0.748	0.763	0.778	0.791	0.802	
63	0.703	0.715	0.723	0.726	0.727	0.728	0.729	0.732
32	0.693	0.708	0.718	0.722	0.724	0.726	0.726	0.722
16	0.678	0.696	0.709	0.715	0.719	0.721	0.722	0.707
8	0.662	0.680	0.695	0.704	0.709	0.713	0.715	0.689
4	0.647	0.661	0.677	0.688	0.696	0.701	0.704	0.674
2	0.636	0.645	0.657	0.668	0.680	0.686	0.690	0.659
1	0.619	0.631	0.643	0.651	0.660	0.668	0.675	0.640

**Table 3:** AUC values for urban scenes.

Nr. of subjects 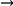 Nr. of images 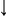	1	2	4	7	13	25	47	Subject-specific
Image-specific	0.731	0.770	0.796	0.813	0.827	0.838	0.846	
63	0.652	0.662	0.667	0.670	0.672	0.672	0.673	0.669
32	0.639	0.652	0.659	0.663	0.665	0.667	0.667	0.657
16	0.623	0.637	0.646	0.652	0.655	0.657	0.658	0.640
8	0.608	0.619	0.630	0.636	0.640	0.643	0.645	0.624
4	0.598	0.605	0.612	0.619	0.624	0.627	0.629	0.612
2	0.593	0.596	0.601	0.604	0.609	0.610	0.612	0.603
1	0.581	0.588	0.592	0.597	0.599	0.600	0.604	0.590

**Table 4:** NSS values for natural scenes.

Nr. of subjects 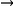 Nr. of images 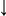	1	2	4	7	13	25	47	Subject-specific
Image-specific	0.741	0.941	1.159	1.319	1.465	1.571	1.638	
63	0.773	0.835	0.871	0.887	0.897	0.903	0.905	0.976
32	0.730	0.804	0.850	0.870	0.882	0.890	0.893	0.929
16	0.664	0.752	0.810	0.837	0.855	0.865	0.870	0.854
8	0.574	0.672	0.744	0.781	0.807	0.822	0.829	0.748
4	0.472	0.570	0.653	0.699	0.732	0.753	0.764	0.623
2	0.368	0.461	0.536	0.593	0.634	0.657	0.666	0.492
1	0.277	0.346	0.439	0.490	0.520	0.559	0.577	0.376

**Table 5:** NSS values for urban scenes.

Nr. of subjects 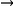 Nr. of images 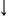	1	2	4	7	13	25	47	Subject-specific
Image-specific	1.020	1.279	1.533	1.708	1.853	1.954	2.013	
63	0.519	0.559	0.581	0.593	0.600	0.604	0.605	0.613
32	0.470	0.519	0.549	0.564	0.572	0.578	0.581	0.559
16	0.403	0.459	0.496	0.515	0.528	0.534	0.538	0.483
8	0.325	0.381	0.425	0.444	0.461	0.473	0.477	0.395
4	0.250	0.303	0.341	0.365	0.382	0.391	0.396	0.307
2	0.186	0.231	0.273	0.284	0.300	0.298	0.305	0.231
1	0.138	0.174	0.195	0.221	0.240	0.230	0.240	0.170

**Table 6:** KL-divergence values for natural scenes.

Nr. of subjects 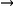 Nr. of images 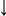	1	2	4	7	13	25	47
Image-specific						0.424	
63	0.900	0.763	0.707	0.684	0.670,	0.662	
32						0.678	
16						0.707	
8						0.757	
4						0.850	
2						1.037	
1						1.467	

**Table 7:** KL-divergence values for urban scenes.

Nr. of subjects 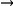 Nr. of images 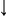	1	2	4	7	13	25	47
Image-specific						0.364	
63	1.274	1.190	1.153	1.141	1.137	1.139	
32						1.153	
16						1.201	
8						1.298	
4						1.501	
2						1.981	
1						3.280	

### Open-source python toolbox

To foster model comparison and ease reproduction of our results we provide a free open-source python toolbox. It allows to conveniently represent fixation data and can be used to estimate the lower and upper bound for fixation selection models on a given data set. Implementations of AUC and KL-divergence, as well as a few other measures, are also contained in the toolbox. The toolbox can be accessed at https://github.com/nwilming/ocupy. Furthermore, the data used in the current work is available from the authors upon request.
